# Supplementary material for: No Significant Association between 25-OH Vitamin D Status and SARS-CoV-2 Antibody Response after COVID-19 Vaccination in Nursing Home Residents and Staff
Source: Vaccines (Basel). 2023 Aug 8;11(8):1343. doi: 10.3390/vaccines11081343 (PMC10458709; doi:10.3390/vaccines11081343)
Supplement: Supplementary file 1 [file vaccines-11-01343-s001.zip › vaccines-2480165-supplementary.pdf]

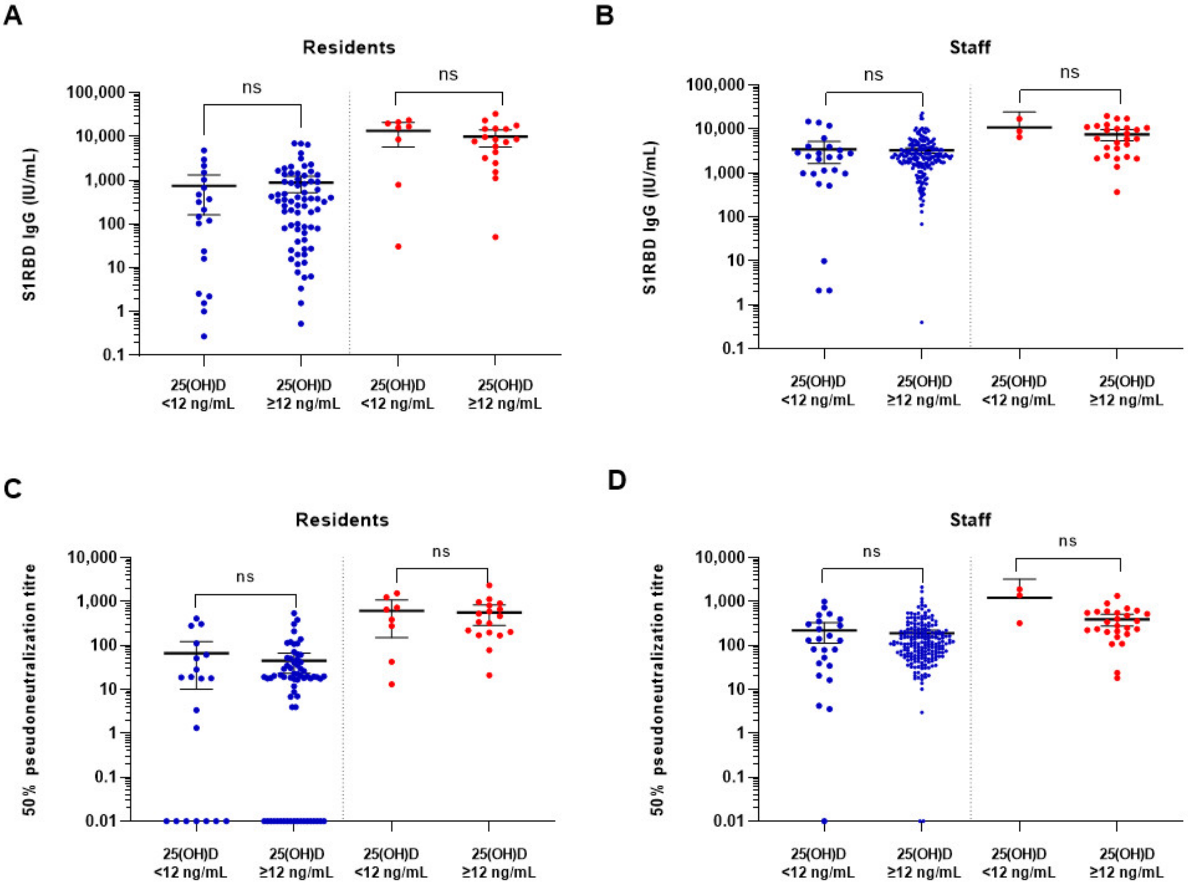

Supplementary Figure S1. S1RBD IgG antibody concentration (International Unit/mL) (**A**,**B**) and 50% pseudoneutralization titer (**C**,**D**) 14 days after BNT162b2 vaccination among nursing home residents (**A**,**C**) and nursing home staff (**B**,**D**) in subjects with serum concentrations of 25-hydroxyvitamin D (25(OH)D) of <12 ng/mL and ≥12 ng/mL as a cut-off for severe vitamin D deficiency. Data is presented stratified for infection naïve participants (blue) and previously infected participants (red). Bold horizontal lines with error bars represent the geometric mean S1RBD IgG antibody concentration and median 50% pseudoneutralization titer per group with 95% confidence intervals. ns: not significant at the 0.05 level. Samples with a 50% pseudoneutralization titer of 0.00 were adjusted to a value of 0.01 in order to plot them on a logarithmic scale.
